# Maternal exposure to childhood maltreatment and adverse birth outcomes

**DOI:** 10.1038/s41598-023-36831-9

**Published:** 2023-06-27

**Authors:** Lauren S. Keenan-Devlin, Ann E. B. Borders, Alexa Freedman, Gregory E. Miller, William Grobman, Sonja Entringer, Hyagriv Simhan, Pathik Wadhwa, Claudia Buss

**Affiliations:** 1 Evanston, IL USA; 2 Department of Obstetrics and Gynecology, NorthShore University HealthSystem, Evanston, USA; 3 University of Chicago Pritzker School of Medicine, Chicago, USA; 4 Institute for Public Health and Medicine, Northwestern University Center for Healthcare Studies, Chicago, USA; 5 Department of Psychology, Northwestern University, Evanston, USA; 6 Institute for Policy Research, Northwestern University, Evanston, USA; 7 Chicago, IL USA; 8 Division of Maternal-Fetal Medicine, Department of Obstetrics and Gynecology, Northwestern University Feinberg School of Medicine, Evanston, USA; 9 Berlin, Germany; 10 Department of Medical Psychology, Charité-Universitätsmedizin Berlin, corporate member of Freie Universität Berlin and Humboldt-Universität zu Berlin, Augustenburger Platz 1, 13353 Berlin, Germany; 11 Development, Health and Disease Research Program, UC University of California Irvine, California, USA; 12 Pittsburgh, PA USA; 13 Division of Maternal-Fetal Medicine, School of Medicine, University of Pittsburgh, Pittsburgh, USA; 14 Irvine, CA USA; 15 Department of Pediatrics, Development, Health and Disease Research Program, University of California Irvine, 1001 Health Sciences Road, Irvine, CA 92697-3950 USA

**Keywords:** Neuroscience, Psychology, Medical research, Risk factors

## Abstract

Exposure to traumatic events during pregnancy may influence pregnancy and birth outcomes. Growing evidence suggests that exposure to traumatic events well before pregnancy, such as childhood maltreatment (CM), also may influence the course of pregnancy and risk of adverse birth outcomes. We aimed to estimate associations between maternal CM exposure and small-for-gestational-age birth (SGA) and preterm birth (PTB) in a diverse US sample, and to examine whether common CM-associated health and behavioral sequelae either moderate or mediate these associations. The Measurement of Maternal Stress (MOMS) Study was a prospective cohort study that enrolled 744 healthy English-speaking participants ≥ 18 years with a singleton pregnancy, who were < 21 weeks at enrollment, between 2013 and 2015. CM was measured via the Childhood Trauma Questionnaire (CTQ) and participants above the moderate/severe cut-off for any of the five childhood abuse and neglect scales were assigned to the CM-exposed group. Common CM-associated health (obesity, depressive symptoms, hypertensive disorders) and behavioral (substance use) sequelae were obtained from standardized questionnaires and medical records. The main outcomes included PTB (gestational age < 37 weeks at birth) and SGA (birthweight < 10%ile for gestational age) abstracted from the medical record. Multivariable logisitic regression was used to test associations between CM, sequeale, and birth outcomes, and both moderation and mediation by CM-related sequelae were tested. Data were available for 657/744 participants. Any CM exposure was reported by 32% of participants. Risk for SGA birth was 61% higher among those in the CM group compared to the non-CM group (14.1% vs. 7.6%), and each subsequent form of CM that an individual was exposed to corresponded with a 27% increased risk for SGA (aOR 1.27, 95% CI 1.05, 1.53). There was no significant association between CM and PTB (9.3% vs. 13.0%, aOR 1.07, 95% CI 0.58, 1.97). Of these sequelae only hypertensive disorders were associated with both CM and SGA and hypertensive disorders of pregnancy did not mediate the association between CM and SGA. Our findings indicate that maternal CM exposure is associated with increased risk for SGA birth and highlight the importance of investigating the mechanisms whereby childhood adversity sets the trajectory for long-term and intergenerational health issues.

## Introduction

The effects of exposure to excess stress on health are well established, and are known to be particularly pronounced when exposure occurs during sensitive developmental windows in early life. Exposure to childhood maltreatment (CM, defined as adverse experiences that occur in childhood such as physical, sexual, emotional childhood abuse, or physicial or emotional neglect) represents among the most pervasive and potent stressors in early life^1^. Population-based surveys in the United States suggest that a considerable proportion of children experience maltreatment, with about 12% reporting abuse or neglect within the past year^2,3^. Globally, lifetime prevalence of CM varies between 8 and 35% depending on maltreatment type and gender^4^.

Individuals exposed to CM exhibit elevated risk for adverse health outcomes during the lifecourse, including cardiovascular, metabolic, and mental disease^5–9^. This notion is based on the concept of developmental origins of health and disease (DOHaD), which postulates that environmental influences such as adversity during sensitive periods of development, when organs are characterized by a high degree of plasticity, can lead to profound and persistent changes in the developing brain and other regulatory systems with significant consequences for an individual’s short- and long-term health^10,11^. Growing evidence from this literature suggests that the effects of CM, particularly among childbearing persons who subsequently become pregnant, may influence the course of their pregnancy, birth and child health outcomes^11–13^. To date, birth outcomes studied in association with CM mainly have focused on length of gestation, demonstrating greater risk of preterm delivery among those exposed to CM^1–3^. There has been less attention on fetal growth restriction, which also poses significant long-term health risks including obesity, diabetes, and cardiovascular disease^4^. Three prior studies have found decreased fetal growth in pregnant individuals with a history of CM^14–16^. However, two of these^15,16^ studies were conducted in small, high-risk cohorts, and none of the three studies account for gestational age at delivery in estimating adjusted birth weight percentiles, thereby calling into question whether the observed associations were specific to fetal growth restriction.

Depression^17–19^, substance abuse^20–22^, obesity^23,24^, and hypertension^25–27^ are common CM-associated sequelae that increase risk for adverse birth outcomes^28–32^ and may thus mediate the association of CM with preterm birth and SGA. It is also possible that these sequelae may moderate these associations because CM-associated variation in maternal-placental-fetal stress biology has been shown to be especially pronounced in the presence of such sequelae^11,33^.

The purpose of this study was three-fold: we aimed to (1) replicate previous reports of associations between maternal CM exposure and adverse birth outcomes (growth restriction and preterm delivery) in a large, representative US sample, (2) examine whether common CM-associated sequelae (depression, substance use, obesity, and hypertensive disorders of pregnancy) mediate the association between CM and adverse birth outcomes, and (3) characterize the potential moderating role of these CM-associated sequelae in the association between CM and birth outcomes.

## Methods

### Study sample

This study uses data from the Measurement of Maternal Stress (MOMS) Study, in which we prospectively enrolled 744 participants from prenatal clinics at four geographically- and racially-diverse US regions (Pittsburgh, PA, Chicago IL, Schuylkill County PA, and San Antonio TX) between June 2013 and May 2015^34–37^. Recruitment was designed to be representative of the sample populations and was powered to examine differences in preterm delivery assuming a rate of 10%. The study included English-speaking participants 18 years and older with a singleton intrauterine pregnancy who were less than 21 weeks pregnant at time of enrollment. Individuals were excluded from participation if they had a known major fetal congenital or chromosomal abnormality, progesterone treatment > 14 weeks of gestation, or chronic corticosteroid treatment (not including inhalers or topical). Institutional Review Boards at each site approved the protocol (Overall IRB Northwestern University #STU00039484 approved 2/13/14), and all participants provided informed consent. All methods were carried out in accordance with relevant guidelines and regulations.

### Procedure

Participants completed questionnaires and self-reported sociodemographic and behavioral characteristics in the second (16.5 ± 2.5 weeks’ gestation) and third (33.7 ± 1.2 weeks’ gestation) trimesters. Pregnancy and clinical outcomes were abstracted from the medical record by trained staff.

### Key variables

Preterm birth (PTB) delivery at < 37 weeks of gestation, calculated from last menstrual period and verified by ultrasound dating, indicated in both the birth parental and neonatal medical record.

Small for Gestational Age (SGA). Infant birthweight, as recorded in the medical record, measuring < 10th percentile for gestational age at birth. Percentile rankings were sex-specific, based on national norms^38,39^.

Childhood Maltreatment (CM) was assessed with the Childhood Trauma Quesitonnaire (CTQ), a widely used instrument to characterize the extent and nature of exposure to abuse and/or neglect before the age of 18^40^. The CTQ measures exposure across five domains: physical abuse; physical neglect; emotional abuse; emotional neglect; and sexual abuse. For each domain, exposure is measured based on responses to five questions with a five-point Likert scale; the total scores within each domain can range from 5 to 25. Standardized cut-off scores can be used to classify exposure levels as being either “none to minimal”, “low to moderate”, “moderate to severe”, or “severe to extreme.” As applied in previous work that utilized the CTQ to classify CM exposure in pregnant individuals and reported associated variation in maternal-placental stress biology relevant for birth outcomes^33,41^, exposure to each domain of CM was defined as the basis of scores indicative of at least “moderate to severe” abuse or neglect. Recognizing that CM varies in type and severity and that these factors may have differential impact upon pregnancy outcomes, we defined CM exposure in 3 ways. First, to characterize overall exposure to any CM, we examined any CM as a binary variable that assigned individuals to the CM group (reference group) if their sum score was 0, and to the CM+ group if the score was >  = 1 (i.e., comparing individuals with less than “moderate to severe” CM to those with at least “moderate to severe” experiences in one or more of the 5 domains). Second, we evaluated exposure to each of the 5 domains of CM separately with the same “moderate to severe” cut off. Finally, recognizing that these individual domains are highly correlated, we assessed cumulative CM, defined as the sum of the number of domains at or above the “moderate to severe” cutoff (range of 0–5), as our exposure variable.

Covariates: The analyses included covariates that might provide alternative explanations for an observed association between CM and either SGA or PTB. Age in pregnancy has a bi-modal association with risk for adverse pregnancy outcomes^42^, and was categorized as ≤ 20, 21–34, and ≥ 35 years. Prevalence of CM as well as adverse pregnancy outcomes are more frequent among Black and Hispanic/Latine individuals^43^, so we assessed ethnicity and race as White, Black, Hispanic/Latine, and ‘Other’. Because in the US CM^44^, PTB^45^, and SGA^46^ are more prevalent in families of low socioeconomic status, we included measures of both childhood socioeconomic status and current socioeconomic status: Childhood poverty, indicating whether the participant’s family received public assistance before the participant’s 18th birthday; and current educational attainment, which is highly correlated with adult income and was coded as high school or less, some college or associates, and bachelor’s degree or more.

CM-associated sequelae as potential mediators or moderators: Potential mediators/moderators included variables from 4 categories that have been reported as common health and behavioral CM sequelae. Mental health was indexed by presence of depressive symptoms in mid pregnancy based on a score of 16 or greater on the CES-D, a widely used tool designed to evaluate depressive symptomatology^32,47,48^. Hypertensive disorders of pregnancy included gestational hypertension, pre-eclampsia, or eclampsia diagnosed during pregnancy and documented in the medical record^25,49^. Any substance use during pregnancy was defined as any alcohol consumption, recreational drug use (marijuana, cocaine, heroin), or smoking since becoming pregnant. (4) Metabolic risk was indexed by an individual’s pre-pregnancy body mass index (BMI, kg/m^2^), categorized as underweight (< 18.5), normal weight (18.5 to < 25), or overweight/obese (≥ 25)^50^.

### Statistical analysis

The associations between the three CM exposure variables and birth outcomes (SGA, PTB) were examined via multivariable logistic regression in separate models, which included ethnicity/race, age, education, and childhood poverty as covariates. Analyses were not adjusted for any of the above-listed CM-associated sequelae because these may be on the potential causal pathway.

Moderation analyses, adjusting for the same covariates listed above, were conducted to evaluate whether CM-associated sequelae modified risk for adverse birth outcomes. For these analyses an interaction term was created between the cumulative CM exposure score, as it best captures variation in CM severity, and each of the 4 potential CM-related sequelae which was included in multiple regression models. Because the moderating influence of the different sequelae may increase if several sequelae are present, we also created a total sequelae score by assigning one point for each CM-associated sequela reported by the participant (range 0–4) and used it in the moderation analyses.

For those CM-associated sequelae that were significantly associated with both CM and SGA or CM and PTB, their mediating effect was tested using PROC CAUSALMED in SAS, which uses a counterfactual approach for mediation analysis^51,52^. Each mediator was considered separately and models were adjusted for ethnicity/race, childhood poverty, age, education, and the other CM-associated sequelae not evaluated as mediators. As CM exposure was evaluated in three different ways (any, cumulative, and domains), a Bonferroni correction was used to account for multiple testing and a p-value of 0.0167 (0.05/3) was used to determine statistical significance. Analyses were conducted using SAS Software Version 9.4.

### Ethics approval and consent to participate

Institutional Review Boards at each site approved the protocol (Overall IRB Northwestern University #STU00039484 approved 2/13/14), and all participants provided consent.

## Results

CM and delivery data were available for 657 of the 744 MOMS study participants. The sociodemographic characteristics of these 657 participants were not significantly different from those in the full cohort (Table 1). The distribution of key study variables and the crude associations between key variables and birth outcomes are summarized in Table 2. 14.6% of study participants reported one form of moderate-to-severe CM, 6.1% reported 2, 6.1% reported 3, 3.0% reported 4, and 1.7% reported all 5 forms of CM. In total, 31.5% reported at least one form of moderate-to-severe CM, which is consistent with the estimated prevalence of CM in the US^53^. The most commonly-reported forms of CM included sexual abuse (18.4%), emotional abuse (16.1%), and physical abuse (13.1%), followed by emotional neglect (9.7%) and physical neglect (8.2%).

**Table 1:** Characteristics of included versus excluded participants.

	Total 744	Excluded 87	Included 657	p
Age				0.21
< 20	41 (5.5)	7 (8.0)	34 (5.2)	
20–29	578 (77.7)	71 (80.5)	508 (77.3)	
30–39	103 (13.8)	10 (11.5)	93 (14.2)	
40+	22 (3.0)	0 (0)	22 (3.3)	
Income (76 missing overall)				0.23
≤ $15,000	108 (14.5)	17 (19.5)	91 (13.9)	
$15,000–50,000	221 (29.7)	19 (21.8)	202 (30.7)	
≥ $50,000	339 (45.6)	39 (44.8)	300 (45.7)	
Missing	76 (10.2)	13 (14.9)	63 (9.6)	
Race and ethnicity				0.07
Black	127 (17.1)	19 (21.8)	108 (16.4)	
White	429 (57.7)	40 (46.0)	389 (59.2)	
Hispanic	145 (19.5)	22 (25.3)	123 (18.7)	
Other	43 (5.8)	6 (6.9)	37 (5.6)	
Married	601 (81.1)	72 (84.7)	529 (80.6)	0.37
Education				0.94
High school or less	198 (26.7)	24 (28.2)	174 (26.5)	
Some college or associates	254 (34.3)	29 (34.1)	225 (34.3)	
Bachelors degree or more	298 (39.0)	32 (37.6)	257 (39.2)	
CESD score	13.7 ± 10.6	15.5 ± 10.1	13.5 ± 10.6	0.12
CTQ score	37.8 ± 14.6	39.2 ± 18.1	37.6 ± 14.2	0.39
Smoking in pregnancy	76 (10.0)	6 (6.9)	70 (10.7)	0.28
Pre-pregnancy BMI	27.8 ± 7.4	27.7 ± 7.3	27.9 ± 7.6	0.84

**Table 2:** Sample characteristics by birth outcomes and any Childhood Maltreatment (CM) exposure.

	Total	non-SGA	SGA	p-value	Term birth (non-PTB)	Preterm birth (PTB)	p-value	No CM reported	CM reported	p-value
	657	594	63		603	54		450	207	
	N (%)	N (%)	N (%)		N (%)	N (%)		N (%)	N (%)	
Sex of the baby				0.18			0.35			0.77
Female	307 (46.8)	272 (45.9)	35 (54.7)		285 (47.2)	22 (42.3)		212 (47.1)	95 (45.9)	
Male	350 (53.4)	320 (54.1)	29 (45.3)		318 (52.6)	32 (61.5)		238 (52.9)	112 (54.1)	
Maternal age (years)				0.39			0.98			0.25
≤ 20	32 (4.9)	29 (4.9)	3 (4.8)		30 (5.0)	2 (3.7)		17 (3.8)	15 (7.3)	
21–29	334 (50.8)	306 (51.5)	28 (44.4)		306 (50.8)	28 (51.9)		228 (50.7)	106 (51.2)	
30–39	269 (40.9)	241 (40.6)	28 (44.4)		247 (41.0)	22 (40.7)		190 (42.2)	79 (38.2)	
≥ 40	22 (3.4)	18 (3)	4 (6.4)		20 (3.3)	2 (3.7)		15 (3.3)	7 (3.4)	
Income (63 missing)				0.99			< 0.01			< 0.01
≤ $15,000	89 (15)	81 (15.1)	8 (14.3)		70 (12.9)	19 (35.2)		45 (11.0)	44 (23.9)	
$15,000–50,000	200 (33.7)	181 (33.6)	19 (33.9)		189 (34.7)	11 (20.4)		133 (32.4)	67 (36.4)	
≥ $50,000	305 (51.3)	276 (51.3)	29 (51.8)		285 (52.4)	20 (37.0)		232 (56.6)	73 (39.7)	
Race/ethnicity				0.45			< 0.01			< 0.01
Non-Latine White	391 (59.7)	359 (60.6)	32 (50.8)		372 (61.9)	19 (35.2)		295 (65.7)	96 (46.6)	
Non-Latine Black	104 (15.9)	93 (15.7)	11 (17.5)		90 (15)	14 (25.9)		64 (14.2)	40 (19.4)	
Hispanic/Latine	123 (18.8)	107 (18.1)	16 (25.4)		109 (18.1)	14 (25.9)		61 (13.6)	62 (30.1)	
Other	37 (5.7)	33 (5.6)	4 (6.4)		30 (5)	7 (13.0)		29 (6.5)	8 (3.9)	
Married	536 (81.7)	483 (81.5)	53 (84.1)	0.6	495 (82.2)	41 (75.9)	0.25	376 (83.7)	160 (77.3)	0.05
Education				0.72			0.35			< 0.01
High school or less	169 (25.7)	150 (25.3)	19 (30.2)		150 (24.9)	19 (35.2)		95 (21.1)	74 (35.7)	
Some college or associates	222 (33.8)	201 (33.8)	21 (33.3)		204 (33.8)	18 (33.3)		142 (31.6)	80 (38.6)	
Bachelors degree or more	265 (40.4)	242 (40.7)	23 (36.5)		248 (41.1)	17 (31.5)		213 (47.3)	52 (25.1)	
Childhood poverty	249 (37.9)	212 (35.7)	37 (58.7)	< 0.01	223 (37.0)	26 (48.1)	0.11	135 (30.0)	114 (55.1)	< 0.01
Depression (CES-D ≥ 16)	209 (31.8)	184 (31)	25 (39.7)	0.16	191 (31.7)	18 (33.3)	0.8	109 (24.2)	100 (48.3)	< 0.01
Hypertensive disorders of pregnancy (HDP)	88 (13.4)	70 (11.8)	18 (28.1)	< 0.01	69 (11.4)	19 (35.2)	< 0.01	52 (11.6)	36 (17.4)	0.04
Alcohol use in pregnancy	55 (8.2)	48 (8.1)	7 (12.5)	0.63	50 (8.4)	5 (5.8)	0.88	38 (8.4)	15 (7.3)	0.62
Smoking in pregnancy	70 (10.5)	59 (9.9)	11 (17.4)	0.07	62 (10.4)	8 (11.5)	0.3	40 (8.9)	30 (14.5)	0.03
Recreational drug use in pregnancy	16 (2.5)	15 (2.5)	1 (1.6)	0.64	14 (2.3)	2 (3.8)	0.51	5 (1.1)	11 (5.3)	< 0.01
Any substance use	123 (18.7)	106 (17.9)	17 (27.0)	0.08	111 (18.4)	12 (22.2)	0.49	79 (17.6)	44 (21.3)	0.26
Body mass index BMI (kg/m2)				0.76			0.02			< 0.01
Underweight (< 18.5)	13 (2)	11 (1.9)	2 (3.2)		10 (1.7)	3 (5.6)		9 (2.0)	4 (1.9)	
Normal weight (18.5–24.9)	287 (43.9)	260 (43.9)	27 (43.6)		271 (45.2)	16 (29.6)		216 (48.2)	71 (34.5)	
Overweight/obese (≥ 25.0)	354 (54.1)	321 (54.2)	33 (53.2)		319 (53.2)	35 (64.8)		223 (49.8)	131 (63.6)	
Childhood Maltreatment (CM)										
Physical neglect	54 (8.2)	42 (7.1)	12 (19.1)	< 0.01	47 (7.8)	7 (13.0)	0.19			
Physical abuse	86 (13.1)	72 (12.1)	14 (22.2)	0.02	77 (12.8)	9 (16.7)	0.42			
Emotional neglect	64 (9.7)	53 (8.9)	11 (17.5)	0.03	57 (9.5)	7 (13.0)	0.41			
Emotional abuse	106 (16.1)	89 (15)	17 (27.0)	0.01	97 (16.1)	9 (16.7)	0.92			
Sexual abuse	121 (18.4)	102 (17.2)	19 (30.2)	0.01	109 (18.1)	12 (22.2)	0.45			
Number of Childhool Maltreatment (CM) sub-scales moderate/severe				< 0.01			0.44			
0	450 (68.5)	416 (70)	34 (54.0)		416 (69)	34 (63)				
1	96 (14.6)	85 (14.3)	11 (17.5)		87 (14.4)	9 (16.7)				
2	40 (6.1)	36 (6.1)	4 (6.4)		36 (6.0)	4 (7.4)				
3	40 (6.1)	34 (5.7)	6 (9.5)		38 (6.3)	2 (3.7)				
4	20 (3)	16 (2.7)	4 6.4)		16 (2.7)	4 (7.4)				
5	11 (1.7)	7 (1.2)	4 (6.4)		10 (1.7)	1 (1.9)				
Adverse birth outcomes										
Small for getational age (SGA)	63 (9.6)				56 (9.3)	7 (13.0)	0.38	34 (7.6)	29 (14.1)	< 0.01
Preterm birth (PTB)	54 (8.2)	47 (7.9)	7 (11.1)	0.38				34 (7.6)	20 (9.7)	0.36

Participants with any history of CM were more likely to have experienced childhood poverty and to have lower educational attainment and income compared to those without exposure to CM. These participants were also more likely to be overweight or obese, use recreational drugs during pregnancy, smoke cigarettes, report symptoms of depression, and develop hypertensive disorders of pregnancy (Table 2).

Sixty three participants (9.6%) delivered SGA infants. Participants who delivered an SGA infant were more than twice as likely to have developed hypertensive disorders during the index pregnancy, and were more likely to have experienced childhood poverty compared to those who did not deliver SGA (Table 2).

Fifty-four participants (8.2%) had a PTB. PTB was more prevalent among participants who were from low-income households (< $15 k/year), identified as Non-Latine Black, had a diagnosis of HDP, and were overweight/obese (Table 2).

### Maternal childhood maltreatment and offspring preterm birth

Having any maternal CM exposure was not associated with PTB in the current study. The PTB rate among participants with CM was 9.7% compared to 7.6% for participants without CM, and this difference was not statistically significant in either crude or adjusted analyses (OR 1.31, 95% CI 0.73, 2.33/aOR 1.07, 95% CI 0.58, 1.97). When using the different types of CM or cumulative CM as a predictor, there was also no significant association with PTB (Table 3).

**Table 3:** Logistic regression results: relationship between small-for-gestational age (SGA) or preterm birth (PTB) and childhood maltreatment (CM).

	Small for gestational age (SGA)				Preterm birth (PTB)			
	Unadjusted OR (95% CI)	p-value	Adjusted^a^ OR (95% CI)	p-value	Unadjusted OR (95% CI)	p-value	Adjusted^a^ OR (95% CI)	p-value
Any childhood maltreatment	1.99 (1.18, 3.37)	0.010	1.61 (0.92, 2.82)	0.097	1.31 (0.73, 2.33)	0.362	1.07 (0.58, 1.97)	0.841
Sum of childhood maltreatment types								
Each additional childhood maltreatment scale moderate/severe	1.36 (1.14, 1.62)	< 0.001	1.27 (1.05, 1.53)	0.016	1.12 (0.90, 1.38)	0.308	1.02 (0.82, 1.29)	0.841
Type of childhood maltreatment								
Physical neglect	3.09 (1.53, 6.25)	0.002	2.35 (1.12, 4.90)	0.023	1.76 (0.76, 4.11)	0.191	1.28 (0.53, 3.11)	0.588
Physical abuse	2.07 (1.09, 3.94)	0.026	1.67 (0.85, 3.31)	0.138	1.37 (0.64, 2.91)	0.417	1.06 (0.48, 2.32)	0.894
Emotional neglect	2.16 (1.06, 4.39)	0.033	1.62 (0.77, 3.41)	0.208	1.43 (0.62, 3.30)	0.407	1.16 (0.48, 2.79)	0.748
Emotional abuse	2.10 (1.15, 3.82)	0.016	1.76 (0.94, 3.30)	0.078	1.04 (0.49, 2.20)	0.912	0.91 (0.42, 1.98)	0.814
Sexual abuse	2.08 (1.17, 3.72)	0.013	1.74 (0.94, 3.21)	0.078	1.30 (0.66, 2.54)	0.452	1.02 (0.50, 2.08)	0.951

^a^Adjusted for covariates: maternal ethnicity/race, maternal age, education, and childhood poverty.

### Maternal childhood maltreatment and small for gestational age infant

**Figure 1:**
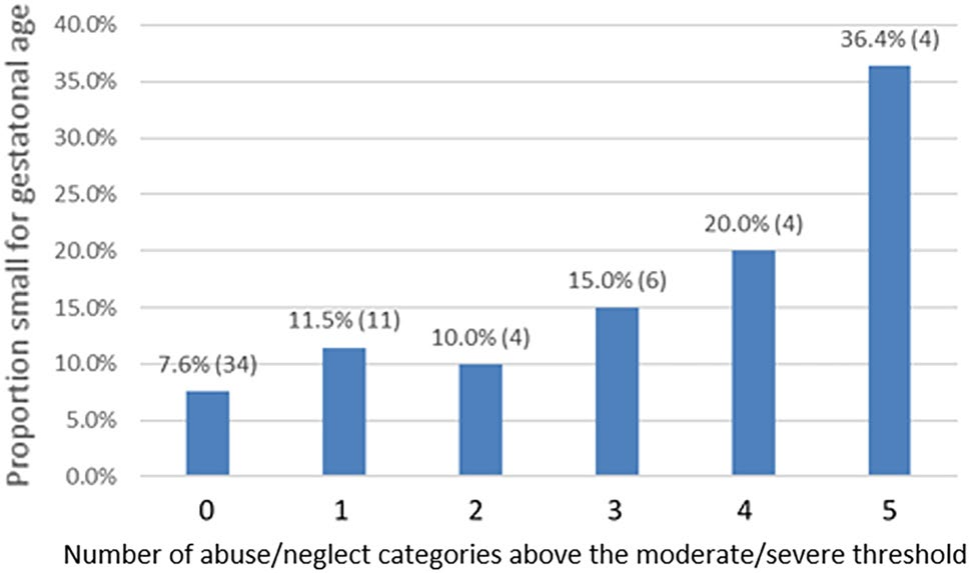
Proportion of small for gestational age (SGA) births by the number of abuse/neglect categories above the moderate/severe threshold.

The prevalence of SGA birth among individuals with any CM was 14.1%, compared to 7.6% among the non-CM group (OR 1.99, 95% CI 1.18, 3.37/aOR 1.61, 95% CI 0.92, 2.82, Table 2). Each of the 5 domains of CM were significantly associated with increased odds of SGA delivery (see Table 2) and cumulative CM exposure was also significantly associated with SGA in both crude and adjusted analyses (OR 1.36, 95% CI 1.14, 1.62/aOR 1.27, 95% CI 1.05, 1.53) (Table 3), even after accounting for multiple testing. In general, as the number of CM domains increased, the proportion of SGA births increased (Fig. 1). When models were adjusted for race/ethnicity, age at delivery, education, and childhood poverty, each subsequent domain of moderate or severe CM endorsed by a participant was associated with a 27% increased likelihood of delivering an SGA infant, compared to subjects who did not report any severe/moderate CM (aOR 1.27, 95% CI 1.05, 1.53) (Table 3).

### CM-associated sequelae as potential moderators of the association between maternal childhood maltreatment and birth outcomes

As depicted in Table 4 none of the 4 CM-associated sequelae or the sequelae sum score moderated the association between CM and SGA, nor that between CM and PTB.

**Table 4:** Results for models of depression, gestational hypertensive disorders, substance abuse, and obesity as moderators of the association between childhood maltreatment (sum score of moderate/severe domains) and adverse outcomes.

	Small for gestational age (SGA)						Preterm birth (PTB)					
	Unadjusted			Adjusted^a^			Unadjusted			Adjusted^a^		
	OR	95% CI	Interaction p-value	OR	95% CI	Interaction p-value	OR	95% CI	Interaction p-value	OR	95% CI	Interaction p-value
Maternal depression risk			0.40			0.34			0.46			0.51
Depressive symptoms	1.24	0.95, 1.61		1.14	0.87, 1.50		1.02	0.74, 1.42		0.95	0.68, 1.33	
No depressive symptoms	1.45	1.13, 1.86		1.37	1.05, 1.78		1.21	0.90, 1.62		1.11	0.82, 1.51	
Obstetric risk			0.22			0.26			0.62			0.65
Hypertensive disorders of pregnancy (HDP)	1.56	1.12, 2.18		1.47	1.03, 2.09		0.99	0.69, 1.41		0.92	0.63, 1.35	
No hypertensive disorders of pregnancy (HDP)	1.22	0.97, 1.53		1.15	0.91, 1.46		1.11	0.81, 1.45		1.03	0.77, 1.37	
Substance use			0.79			0.58			0.56			0.91
Any substance use	1.30	0.92, 1.81		1.15	0.80, 1.66		1.23	0.83, 1.84		1.04	0.67, 1.59	
No substance use	1.37	1.11, 1.68		1.29	1.04, 1.61		1.07	0.83, 1.38		1.01	0.77, 1.31	
Obesity			0.68			0.50			0.46			0.56
Overweight (BMI^b^ ≥ 25)	1.38	1.11, 1.72		1.29	1.03, 1.62		1.04	0.81, 1.34		0.99	0.76, 1.27	
Normal weight/underweight (BMI^b^ < 25)	1.26	0.87, 1.82		1.11	0.75, 1.64		1.26	0.81, 1.95		1.16	0.72, 1.86	

^a^Models adjusted for ethnicity/race, maternal age, education, childhood poverty. ^b^BMI = Body Mass Index weight/height^2^.

### CM-associated sequelae as potential mediators of the association between CM and SGA

Of the CM-associated sequelae, only hypertensive disorders was significantly associated with CM and SGA (Table 2) and was therefore tested as a potential mediator. As shown in Table 5, hypertensive disorders did not significantly mediate the relationship between CM and SGA.

**Table 5:** Results for models gestational hypertensive disorders as a mediator of the association between childhood trauma (score of moderate/severe domains) and small for gestational age birth (SGA).

	Total effect OR (95% C.I.)	Direct effect OR (95% C.I.)	Indirect effect	% Mediated (95% CI)
Mediator of interest				
Hypertension^a^	1.21 (0.95, 1.47)	1.18 (0.93, 1.43)	1.03 (0.99, 1.07)	14.7 (− 5.9, 35.3)

^a^Model adjusted for ethnicity/race, maternal age, BMI, education, childhood poverty, substance use, and depression.

## Discussion

The present results replicate the previously-observed association between CM and risk of an SGA birth, and indicate that CM is associated with a 61% overall increased risk of SGA. CM may be a significant clinical risk factor for SGA birth, as the association was comparable to that of established risk factors^54^ including older age in pregnancy (aOR 1.4^55^), hypertensive disorders of pregnancy (RR 1.0–4.8^56^), nulliparity (aOR 1.3–2.1^57^), and smoking during pregnancy (aOR 2.41^57^). Additionally, we demonstrated a dose–response association between number of abuse/neglect categories experienced during childhood and likelihood of an SGA delivery, with the risk of SGA birth increasing by 27% for every additional form of severe/moderate abuse/neglect category reported. The prevalence of CM in our sample was consistent with US epidemiological data^53^, though we found higher rates of sexual abuse and lower rates of neglect in this sample than expected. The 8.1% of PTB rate in this study was slightly lower than the national average for that same period (9.6%), and the 9.6% SGA rate was similar to that of the US overall (10%)^58^. In this healthy sample that was not enriched for CM exposure, elevated risk for SGA did not appear to stem from depression, substance use, obesity, or hypertension associated with CM exposure. We were unable to replicate a significant association between CM and PTB which has been demonstrated, albeit inconsistently, in prior studies^13,59^. Unlike those studies, the MOMS cohort was inherently at lower risk for PTB due to the exclusion of individuals on progesterone treatment for prior preterm birth, so this may in part explain our findings.

These findings extend the literature in a number of ways. First, we replicated CM and growth associations previously detected in small relatively disadvantaged cohorts^15,16^ in a large US sampleStevens-Simon and McAnarney 1994 included 127 low-income Black adolescent individuals from Rochester NY^16^, and Gavin et al. 2011 examined a cohort of 136 deliveries from Seattle, WA with greater than 50% childhood and adult poverty rates^15^.

Second, while SGA and PTB incidence overlap, our results suggest that CM may impact fetal growth and length of gestation differentially. Only three prior studies have investigated fetal growth as an outcome, and these studies examined low birthweight but not growth percentile^14–16^, which does not identify growth abnormalities per se, since it combines consequences of PTB with those of growth restriction. By differentiating growth effects from length of gestation in our outcomes variables, our analysis indicates that these outcomes may result from different pathways.

Third, our results suggest a CM and SGA association independent of CM-related sequelae. In concordance with previous studies we observed that depression, substance use, obesity, and gestational hypertensive disorders were significantly more common among individuals exposed to any form of CM, compared to those with no CM exposure. Of these, only gestational hypertensive disorders was more common among those delivering SGA infants, but gestational hypertensive disorders did not mediate nor moderate the association between CM and SGA. Substance use has previously been reported to mediate associations between CM and both birthweight and PTB^15,16^. However, these study populations had considerably higher rates of prenatal tobacco, alcohol, and recreational drug use (29% in Simon and McAnarney 1994 and 21% in Gavin et al. 2011) than our cohort^15,16^. The prevalence of substance use in our cohort may have been insufficient to exhibit a significant association with either birthweight or length of gestation.

Our results add to a growing literature evidencing the importance of early life exposures on intergenerational health outcomes. Adverse experiences during this sensitive developmental period may induce permanent or long-term alterations in neural, endocrine and immune systems which may be adaptive from the perspective of increased chances of survival in the short term. Over time these alterations may, however, have deleterious or unfavorable effects on the health and well-being of an individual^72^ and future generations^11^. CM-associated variation in maternal-placental-fetal (MPF) stress biology may underlie the observed association between CM and SGA^60^. CM has been associated with variation in cortisol concentrations, steeper increases in placental CRH and increased systemic inflammation during pregnancy (reviewed in Moog et al.^11^), biological alterations that may impair fetal growth by leading to maternal vascular underperfusion and/or chronic placental inflammatory lesions that reduce placental function critical for fetal growth^61^. Reduced intrauterine growth in association with maternal CM may contribute to higher cross-disease morbidity in offspring^62^, starting with the effects on fetal growth and development^1–3,14–16^ and extending to subsequent infant, child (behavioral problems, autism), and even adult health and disease risk^63,64^.

While prevalence rates for the other sequelae were in concordance with reported national rates^65–67^, our sample size (and consequent number of SGA deliveries in this sample) was too small to identify mediators of the association between CM and this complex phenotype with multifactorial etiology. We suggest that future studies take advantage of cohorts enriched for SGA births to replicate the higher prevalence of CM among individuals delivering an SGA offspring and investigate the role of these sequelae as potential mediators.

In the context of a healthy population not enriched for CM, our findings point to the potential clinical significance of screening for CM to detect risk for growth restriction and other adverse outcomes. The CTQ has been validated in multiple iterations that may be clinically useful^68^, and by identifying individuals exposed to CM, may provide opportunity for therapeutic intervention during pregnancy. Moreover, these findings indicate the need to identify effective treatments, such as those that may reduce stress and normalize stress physiology, which as a consequence may reduce SGA risk among those with CM histories.

This study has some limitations. First, CM was based on retrospective self-report using the CTQ. Retrospective reports in adulthood of childhood adverse experiences are subject to problems like non-awareness, non-disclosure, simple forgetting, and reporting biases due to mood states. However, while the recall of subtle details of early traumatic experiences may depend upon personal interpretations and more prone to recall bias, major experiences (i.e., abuse) are better recalled. In general, false negatives are more frequent than false positives^69^. This downward bias should, therefore, result in an observed estimate of the association between CM and SGA during pregnancy^70^ that is more conservative than the one that actually exists. Additionally, the CTQ demonstrates high internal consistency, good test–retest reliability, and convergence with other instruments^65^. The CTQ does not collect the date of exposure to CM, so we were unable to examine whether the timing of abuse is relevant to the association of CM and obstetric outcomes. We were also unable to evaluate PTSD symptomology or diagnosis, another highly prevalent consequence of CM, which in future studies should be examined as a potential mediator or moderator. Smoking and substance use during pregnancy was also based on self report, which is typically underreported^66^ and could have obscured this pathway in our study. Finally, we are not able to comment on resilience factors that may have contributed to these findings.

Our findings extend those from previous studies of childhood adversity and birth outcomes^14,34,67^ to show that CM is associated with increased risk for SGA birth independent of substance use, depression, obesity, and hypertensive disorders during pregnancy. These findings support the notion that adversity in childhood sets a trajectory for long-term and intergenerational patterns of health, and may underlie persistent population disparities^71^.

## Data Availability

The datasets used and/or analysed during the current study are available from the corresponding author on reasonable request.
